# Intervention mapping to address social and economic factors impacting indigenous people’s health in Suriname’s interior region

**DOI:** 10.1186/s12992-017-0238-4

**Published:** 2017-03-01

**Authors:** Daniel Peplow, Sarah Augustine

**Affiliations:** 1 Department of Health Services, University of Washington, White Swan, WA USA; 2 Suriname Indigenous Health Fund, White Swan, WA USA; 3 Sociology Department, Heritage University, Toppenish, WA USA

**Keywords:** Indigenous health, Economic policy, Global health governance, Multi-lateral development banks

## Abstract

**Background:**

Previous studies found that while internationally financed economic development projects reduced poverty when measured in terms of per capita GDP, they also caused indigenous people to become disassociated, impoverished and alienated minorities whose health status has declined to unacceptable lows when measured in terms of mercury poisoning and the burgeoning rate of suicide. In this study, we developed a needs assessment and a policy-oriented causal diagram to determine whether the impaired health of the people in this region was at least partially due to the role the country has played within the global economy. Specifically, could the health and well-being of indigenous people in Suriname be understood in terms of the foreign investment programs and economic development policies traceable to the Inter-American Development Bank’s *Suriname Land Management Project*.

**Methods:**

Interviews took place from 2004 through 2015 involving stakeholders with an interest in public health and economic development. A policy-oriented causal diagram was created to model a complex community health system and weave together a wide range of ideas and views captured during the interview process.

**Results:**

Converting land and resources held by indigenous people into private ownership has created an active market for land, increased investment and productivity, and reduced poverty when measured in terms of per capita GDP. However, it has also caused indigenous people to become disassociated, impoverished and alienated minorities whose health status has declined to unacceptable lows.

While the effects of economic development programs on the health of vulnerable indigenous communities are clear, the governance response is not. The governance response appeared to be determined less by the urgency of the public health issue or by the compelling logic of an appropriate response, and more by competing economic interests and the exercise of power.

**Conclusion:**

The health and well-being of the indigenous Wayana in Suriname’s interior region is at least partially due to the role the country has played within the global economy. Specifically, the health and well-being of indigenous people in Suriname can be understood to be a result of foreign development bank-funded projects that drive the government of Suriname to trade land and natural resources on the global market to manage their country’s balance of payments.

## Background

Many of the causal determinants of health and health inequities lay outside the health sector and are socially, economically and politically formed [1]. Economic policies, promoted by international agencies and triggered by free-trade agreements and globalization, have resulted in a proliferation of large-scale development projects on indigenous lands and territories [2–7]. International banking institutions provide structural adjustment loans and fund infrastructure projects that are necessary for resource extraction and export. For the U.S., the Department of Treasury leads the Administration’s engagement in the multilateral development banks (MDBs), which include the World Bank, Inter-American Development Bank (IADB), Asian Development Bank, the African Development Bank, and the European Bank for Reconstruction and Development [8].

MDBs provide loans and low interest subsidies on the condition that the developing country agrees to adopt Structural Adjustment Programs (SAPs) that remove excess government controls and promote market competition consistent with the neo-liberal ideology that drives globalization [9–11]. The aim of SAPs is to achieve long-term or accelerated economic growth in poorer countries by restructuring their economies and reducing government intervention. SAP policies include the managed balance of payments and the reduction of government services through public spending cuts and budget deficit cuts, the privatization of non-market land and natural resources, increased free trade, and business deregulation. Governments of developing countries are forced to open up their economies to foreign direct investment (FDI) and reduce their role in the economy by privatizing the health sector as well as state-owned industries and non-market land and natural resources [7, 12–16].

Simple trade models suggest that developing countries should specialize in industries in which they have a comparative advantage to manage their balance of payments, which is a record of international transactions that balance net earnings on exports minus payments for imports [7]. In general, the structure of balance of payments is a reflection of the degree to which the Suriname economy relies on the outside world for goods and services it imports as well as its capacity to pay for these using its export proceeds. Following this model, countries like Suriname that are rich in natural resources specialize in the extraction of those natural resources so they qualify for subsequent installments of what amounts to collateralized loans that are divided into segments marked by milestone payments. The booming sector becomes the extraction of natural resources. In Suriname, mining is the largest income generating segment of the economy [7, 14].

To manage their balance of payments, Suriname responded by increasing its capacity in the exploration and exploitation of gold and bauxite [7]. It is generally recognized that most of the funds obtained by Suriname are in the form of loans from the IADB, of which there have been 198 since 1982 [7, 17]. This implies that the development process is inevitably accompanied by indebtedness. Indigenous peoples, who are dispossessed of the territories they occupy and rely upon for their traditional livelihoods, become dislocated, scattered, impoverished and alienated minorities by the SAPs that comply with neoliberal economic development programs [16]. They also become frustrated by the persistent disjuncture between their need to address the causes of health and well-being that are socially and economically formed and the failure of their government to respond. What generally escapes the world’s attention is the funders’ influence on the development strategy and policies of the recipient, in this case Suriname. Given that the IADB is in a stronger position than Suriname, the Government of Suriname (GOS) is forced into an extremely weak, vulnerable and dependent position [7]. It is a process that is currently benefitting some by increasing GDP per capita at the expense of vulnerable minority Tribal and Indigenous People who reside on the areas being privatized.

While converting land and resources held by indigenous people into private ownership has created an active market for land, increased investment and productivity, and reduced poverty when measured in terms of per capita GDP, it has also caused indigenous people to become disassociated, impoverished and alienated minorities whose health status has declined to unacceptable lows when measured in terms of mercury poisoning and the burgeoning rate of suicide [18–25].

The first country cooperation framework (CCF) for Suriname outlined a strategy for economic reform, and the UNDP on behalf of the international donor community outlined the supporting policies needed to create an enabling environment that would enhance Suriname’s productive capacity. The GOS asked the UNDP, in collaboration with UNIDO, to take a lead role in combating the Hg pollution which results from gold mining [13, 14]. A 2015 World Wildlife Fund review on mercury contamination from small-scale gold mining in Suriname showed that in the last 20 years, while SAPs have led to significant economic improvements, they have failed to limit Hg pollution which has become a significant environmental and public health issue across the nation and in the region [26]. The authors of the report recommended that measures be taken to address the effects of Hg exposure among Suriname’s most vulnerable people living in the interior region.

Concerns about the ecological effects of mining, and of exposure to Hg from mining, increased in 2001 when Mohan carried out a pilot study on the exposure to Hg in mothers and newborns who were seen in the obstetrics and gynecology ward of a hospital in Suriname’s capital [27]. Between 2005 and 2012, Peplow and Augustine showed mercury exposure was also occurring in Suriname’s interior region at levels causing adverse neurological effects among the indigenous Wayana people [28–30] who are highly dependent on fish in their diet [31]. Acknowledging the extreme nature of the Wayana health crisis compelled public health practitioners to address the large-scale social forces at work [32].

While the attention paid to technological and behavioral solutions at the individual level yields important health outcomes, attention should also be paid to structural causes of disease, disability and premature death [33]. In this paper we describe the use of community-led public health research to highlight the link between international development bank policy and practice and community health. The focus of our attention is away from discrete national systems to a study of how these systems are influenced and shaped by internationally financed economic development loans and programs.

In this decade-long project, we tested the hypothesis that the causes of health and well-being among the indigenous people in Suriname’s interior ‘Amazonia’ region lie outside the health sector and are socially and economically formed and that their health and well-being is linked to globalization and ‘governed’ on a global scale. The underlying assumption is that there is a complex web, or system of causation that provides a context for intervention. We assume that identifying the most effective leverage points within this web, in which small changes in the social environment can lead to large changes in health outcomes, is essential to reduce complexity and develop effective, multilevel intervention plans.

The IADB’s Suriname Land Management Program (SLMP) is a useful case example of how economic development programs that convert non-marketed land and mineral resources into marketable resources for the global economy can affect health outcomes. As of 2004, when this project began, between 10 and 15 million people worldwide, including approximately 3 million women and children, were utilizing Hg in artisanal and small-scale gold mining operations [26]. Our goal was to support the original “people-centered” strategies for economic-development that meets the “balance-of-payment” goals as described in the UNDP 1997–2002 Advisory Note on the Country Cooperation Framework of Suriname [14] and is reflected in the Inter-American Development Bank’s 2006 Operational Policy on Indigenous Peoples and Strategy for Indigenous People [33]. We expect the lessons learned in Suriname can be applied to other areas experiencing similar issues.

## Methods

This paper took a systems approach to intervention mapping to identify causal relationships and to select the most effective leverage points that address health related problems at the community level [34]. The systems approach was used to create a social-ecological model in which health conditions could be viewed as a function of the interaction of individuals with the environment in which they live as opposed to a linear model focusing on a single cause-effect pathway. Health, as it related to individual behavior, was viewed as a function of factors found in their environment, including family, social networks, organizations, communities, government and supranational organizations.

Bartholomew outlined six fundamental steps in the intervention mapping process [34]: 1) Identify problems and assess causes, 2) Identify changeable determinants, 3) Select theory-based methods to change determinants, 4) Create an intervention action plan, 5) Implement intervention action plan, and 6) Review, reflect and evaluate intervention program.

Even though the process is described as a series of steps, the process we followed was iterative, not completely linear and followed a progressive cycle of planning, action and reflection that was carried out continually over the 7-year period during which this project was performed. The intervention mapping process spanned ecological levels starting at the individual level and proceeded through the interpersonal, community, and societal levels. This approach relied on horizontal relationships between various partners through a democratic participatory process. It was built on a broad base of relationships in which various types of knowledge were brought together to illuminate issues identified by indigenous communities impacted by specific economic development projects.

### Theory

In this intervention mapping study, we used theory to guide the planning process and protect against type-III error, that is, an improperly designed intervention action plan that included correctly identified problems that should not actually be addressed because significantly large improvements in health outcomes would not be achieved by making small changes in the environment. Multiple theories can be used to assess, intervene, solve and prevent the causes of health and well-being that lie outside the health sector and are socially and economically formed [32]. The main focus was on problem solving and the criteria for success were defined in terms of the problem rather than the theory. For that reason, the problem-driven approach required that this program be informed by multiple theories to address problems at the interface between health, well-being and economic development. The theories used to ensure the intervention map adequately identified and described factors that could be addressed through reasonable actions to achieve adequate health outcomes are shown in Table 1.

**Table 1:** Theories used to guide the planning process and protect against type-III error, that is, an improperly designed intervention map that includes correctly identified problems that should not actually be addressed because significantly large improvements in health outcomes would not be achieved by making small changes in the environment

	Theory (ref. no.)	Principle	Application	Desired Outcome
1	Systems Theory [38, 50–52, 57–59]	Describes a nested structure of factors affecting health including physical, social and cultural. What emerges is a nested structure of environments that allows for multiple influences both vertically across levels and horizontally within each level. This complex web or system of causation is a rich context for intervention.	Applied to the political and economic system, legal framework, enforcement agencies, established patterns of social organization, public administration, and demographics. Also, the many potential combinations of educational, social, political, regulatory and organizational supports to improve health.	Used to provide framework for mapping relationships between stakeholders, reduce complexity and look for the most effective leverage points within this web in order to develop effective multilevel interventions.
2	Social Network Theory [60]	Describes social networks that consist of nodes (individuals, groups, or organizations) and are joined by ties (relationships among nodes). A community is a network of networks in which the nodes of the larger network comprise smaller-scale networks.	Applied to the political and economic system, legal framework, enforcement agencies, established patterns of social organization, public administration, and demographics. Also, the many potential combinations of educational, social, political, regulatory and organizational supports to improve health.	Used to engage stakeholders based on their potential to secure benefits by virtue of membership in social networks or other social structures. The Social Network approach was also used to reduce complexity and look for the most effective leverage points within this web of causation to develop opportunities for effective multilevel interventions.
3	Stakeholder Theory [61]	Acknowledges stakeholders who differ in their social, political, and ethical characteristics; goals, interests; and types and amounts of power. Health promoters, their organizations, and the communities with which they work are frequently external stakeholders and exist outside the “focal organization” but have a direct interest in what that organization does.	Applied to the political and economic system, legal framework, enforcement agencies, established patterns of social organization, public administration, and demographics. Also, the many potential combinations of educational, social, political, regulatory and organizational supports to improve health.	Used to identify, map, and bring together stakeholders who may differ from each other in their social, political, and economic goals and interests and types and amounts of power.
4	Empowerment Theory [62]	Describes how to transfer power (a process) and the consequences of that process (an outcome). Empowerment Theory assumes that when health problems revolve around relational power processes then who holds power and how it is exercised can be used to guide health intervention strategies.	Applied to marginalized communities undergoing assimilation into dominant market driven societies.	Used to create a new social contract between health and other sectors to advance human development, sustainability, and equity, as well as improve health outcomes. Reduce inequalities and social gradients to improve health and well-being for everyone.
5	Community Participation Theory [63–66]	Describes a complex and context specific approach that seeks to maximize the benefits of social relationships and the efficient use of social capital. Social capital can be placed at the individual level, the community level or societal level.	Applied to situations where it is necessary to overcome difficulties imposed by a lack of consent or engagement by disenfranchised communities that discourage the creation of new knowledge in neglected areas of health.	Used to engage and include marginalized and disadvantaged populations, empower people, mobilize resources and energy. Also used to develop holistic and integrated approaches to public health problems. Achieve better decisions and more effective services and ensure the ownership and sustainability of programs.
6	Grassroots or Community Organizing Theory [66, 67]	Describes an approach to policy change that is made through collective action by members of the community addressing problems affecting their lives. Leadership is provided by a distinct group of individuals directly affected by an issue. Public health practitioners act as “conveners” or in a “capacity-builder” role rather than the “driver” role.	Applied to situations where it is necessary to overcome difficulties imposed by a lack of consent or engagement by disenfranchised communities that discourage the creation of new knowledge in neglected areas of health.	Used to increase democracy as it applies to health. Combat exclusion of marginalized and disadvantaged populations. Empower people, mobilize resources and energy. Develop holistic and integrated approaches to public health problems. Achieve better decisions and more effective services and ensure the ownership and sustainability of programs.
7	Advocacy Theory [67]	Describes actions that can be taken to bring about change on behalf of another population. Public health advocacy, often confused with activism, is rooted in democratic principles and practices and includes cooperation as well as confrontation.	Essential when working with communities undergoing assimilation when acculturation has taken place but institutional assimilation has not or is incomplete.	Advocacy ensures that the rights of disenfranchised individuals are protected, that institutions work the way they should, and that legislation and policy reflect the interests of the people.
8	Media Advocacy [68, 69]	Describes a set of tactics and the strategic use of the media to support community organizers’ efforts to advance social or public health policies.	Targets policy makers and those who can be mobilized to influence them since they can control the environments that either promote health or foster disease.	Used as a forum to surface issues, identify topics for discussion, and set the agenda for policymakers and the public.
9	Agenda Building Theory [38]	Defines issues that merit active and serious consideration by political decision and policy makers. Agenda building is the process of moving an issue to the systemic and institutional agenda for action.	Applied using the outside-initiative model to policy makers and those who can be mobilized to influence them since they can control the environments that either promote health or foster disease.	Used to develop strong high-level policy processes at the interface between health, well-being and economic development.
10	Multiple Streams Theory [71]	The Multiple Streams Theory distinguishes between seperate discourses that determine global health, e.g. biomedicine, public health, economism, human rights, security.	Applied to situations in which the determinants of health and well-being lie outside the health sector and are socially and economically formed.	Used to create a new social contract between health and other sectors to advance human development, sustainability, and equity, as well as improve health outcomes. Reduce inequalities and social gradients to improve health and well-being for everyone.
11	Consequentialist Theory [69]	This theory judges the rightness or wrongness of an action based on the consequence that action has. In contrast, non-consequentialist theory judges an action based on the properties intrinsic to the action, not its consequences.	It could be argued that when applied to economics the SLMP satisfies the criterion for being right according to the consequentialist theory if it benefits the greater good and harms only a small number of people.	The implication for human rights is that even though the SLMP provides a benefit to a great number of people, the health and well-being of indigenous communities, which is a protected human right, will always trump economic development.

In this study, the use of theory in performing evidence-based intervention mapping was not about the methods used, but the methodological context of their application. While research is often designed to generate knowledge for understanding, the purpose of this project, motivated by pragmatism and concerns of equity, was to generate knowledge for action. The aim of the methodologies used were to engage and take advantage of the knowledge and experience of individuals in institutions ranging from small community organizations and NGOs to national and international organizations.

### First-person narrative accounts from meetings with stakeholders across sectors and disciplines

The discussions were carried out with stakeholders that had a direct interest in development, health or the SLMP (Table 2). Meetings were held to discuss why there is such a marked disjuncture between the health needs of people displaced by economic development, and by extension their global health needs, and governance responses. Communication between stakeholders, irrespective of their discipline or background, was accomplished using self-narration as an empirical resource, as discussed by Patel who argued that self-narratives are evidentiary and meaningful as testimony [35]. Other data were obtained throughout the project such as unpublished internal reports and other materials collaborators provided.

**Table 2:** Stakeholders interviewed to create the Intervention Map

Key	Commentator
	Communities
1	Kawemhakan (Anapayke), Lawa River, Sipaliwini District, Suriname
2	Commisaris Kondre, Saramacca River, Brokopondo District, Suriname
3	Makki Kriki, Saramacca River, Brokopondo District, Suriname
4	Puleowime (Apetina), Tapanahony River, Sipaliwini District, Suriname
5	Kawemhakan (Anapayke), Lawa River, Sipaliwini District, Suriname
6	Antecume Pata, Maroni River, French Guiana
7	Twenke, Maroni River, French Guiana
	Community Coalitions
8	Coordinator of Indigenous Organizations of the Amazon River Basin (COICA)
9	Organization of Indigenous People in Suriname (O.I.S.)
	Suriname
10	Political and Economics Section Chief, US Embassy Paramaribo, Suriname
11	Missionary, World Team Suriname, Apetina, Suriname
12	Director, World Wildlife Fund, Paramaribo, Suriuname
13	Country Representative, Pan American Health Organization, World Health Organization, Paramaribo, Suriname
14	Environmental Health Advisor, Pan American Health Organization, World Health Organization, Paramaribo, Suriname
15	Director, National Insititute for Environment and Development in Suriname (NIMOS), Paramaribo, Suriname
16	Director, Physician, Medical Laboratory, Paramaribo, Suriname
17	Director, Primary Health Care Suriname (MZ), Paramaribo, Suriname
18	Operations Specialist, Inter-American Development Bank, Paramaribo, Suriname
19	Attorney, Paramaribo, Suriname
20	Head Medical Office, Ministry of Labour, Technological Development and Environment
21	Chief of Political-Economic Section, Embassy of the United States of America, Paramaribo, Suriname
22	Community Relations, Public Communications Coordinator, Canadian-based Gold Mining Corporation, Paramaribo, Suriname
23	Managing Director, Private Outdoor Guide Service, Paramaribo, Suriname
	United States
24	Suriname Country Desk Officer, US Department of State, Washington, D.C.
25	Information Officer, Pan-American Health Organization, Washington, D.C.
26	Health Communication Officer, Pan-American Health Organization, Washington, D.C.
27	Consejero Principal, Guyana, Jamaica y Trinidad y Tabago, Inter-American Development Bank, Washington, D.C.
28	Planning Economist, Ministry of Planning and Development, Inter-American Development Bank, Washington, D.C.
29	Managing Director, Amazon Team, World Wildlife Fund, Washington, D.C.
30	Conservation Director, Guyanas, World Wildlife Fund, Washington, D.C.
31	Chief of Staff, Assistant Secretary General, Organization of American States, Washington, D.C.
32	Senior Human Rights Specialist, Inter-American Commission on Human Rights, Organization of American States, Washington, D.C.
33	Social Development Specialist, Environmental Protection Unit, Interamerican Development Bank, Washington, D.C.
34	Senior Country Officer, World Bank, Washington, D.C.
35	Constituent Services Representative, US Senate, Washington, D.C.
36	Consulting Physicians on Risk and Health Assessment Delegations to Indigenous Communities
	International
37	UN Special Rapporteur on the Rights of Indigenous Peoples, United Nations Human Rights Council (UNHRC)
38	Rapporteur on the Rights of Indigenous Peoples, Inter American Commission on Human Rights, Organization of American States
39	Coordinator Health Promotion, World Health Organization, Geneva, Switzerland
40	Executive Director, Commercial Bankers, Board of Governors, Inter American Development Bank
39	The International Bank for Reconstruction and Development/The World Bank, Civil Society Policy Forum

Cognitive Mapping techniques were also used as guides to integrate narrative results, generate cross-disciplinary links, and assess mental models with an emphasis on each individual’s ownership of the concepts that serve as the landmarks in their own cognitive maps [36]. The cognitive mapping technique was used to aid the interviewing process and evaluate policy, program and project effectiveness.

Open-ended interviews were used to study people’s mental models and to determine how four specific factors relating governance, economic development and indigenous community health are viewed:

1. *The conversion of land and resources held by indigenous people into private ownership has caused indigenous people to become disassociated, impoverished and alienated minorities whose health status has declined to unacceptable lows when measured in terms of increased death, disease and disability.*
2. *Development strategies should be people-centered and include improved governance, poverty alleviation and sustainable livelihood generation with a specific focus on displaced indigenous communities, the establishment of improved social services, and the translation of gains in the macro-economic sphere into concrete benefits for the entire population with a special emphasis on the most vulnerable groups.*
3. *Indigenous people living in “isolation” and “trans-border” Indigenous Peoples living in territories that straddle two or more countries (*e.g. *The Wayana in Suriname, French Guiana, and Brazil) who are vulnerable to the integration process are the most vulnerable and should be given preference.*
4. *Structural Adjustment Programs (SAPs) of the past four decades should be recast to provide land tenure to indigenous communities so communities undergoing assimilation and acculturation can join the market economy and transform their land assets into sustainable livelihoods.*

Interviews were conducted in three steps: 1) participants were introduced to the four factors described above relating governance, economic development, and indigenous health; 2) participants were asked to explain their view of each issue and how they relate to the other components; 3) as the components and their relationships were captured and described, the interviewer arranged the components on the evolving’ policy-oriented causal diagram’ until it illustrated how the participant perceived the issue.

### Cause-and-effect

Inferential criteria for use in environmental toxicology studies were used to establish a cause-effect relationship between system-level processes and the health and well-being of impacted communities [37].

### Agenda setting

This study used the outside initiative model (OIM) to expand public health issues, which originate in the civil society sector, and extend them to the public sector and ultimately place them on the formal political agenda for resolution [34, 38]. This project used the OIM as a guide to search for cooperative solutions across sectors at the policy level and to facilitate more equitable patterns of growth and development leading to measurably improved health outcomes.

### Site

Suriname is a small country in South America, lying north of Brazil, between Guyana and French Guiana. As of 2009, the population of Suriname was approximately 524,143 people [39]. There are approximately 500 Wayana people living in Suriname, and a total of about 1500 Wayana living throughout Suriname, Brazil and French Guiana [40], an area known as the Guiana Region.

### Ethical considerations

The primary intent of the work discussed in this paper was aimed at a specific public health problem: to support the indigenous people in Suriname in their efforts to self-diagnose public and environmental health problems from exposure to Hg contamination [28–30]. This paper addresses the secondary benefits of the community-led efforts at the interface between health, well-being and economic development. These public health intervention projects were reviewed by the University of Washington Institutional Review Board (IRB), Human Subjects Division (HSD). No reference number was assigned because the community-led project was conducted as a public health service and was deemed “non-research”. Consequently, the work was not considered to be within the purview of institutional review. As such, these non-research investigations have yielded insights of generalizable value that merit dissemination, but the research versus non-research determination, which is based on the primary intent, remains unchanged [41].

## Results

**Fig. 1:**
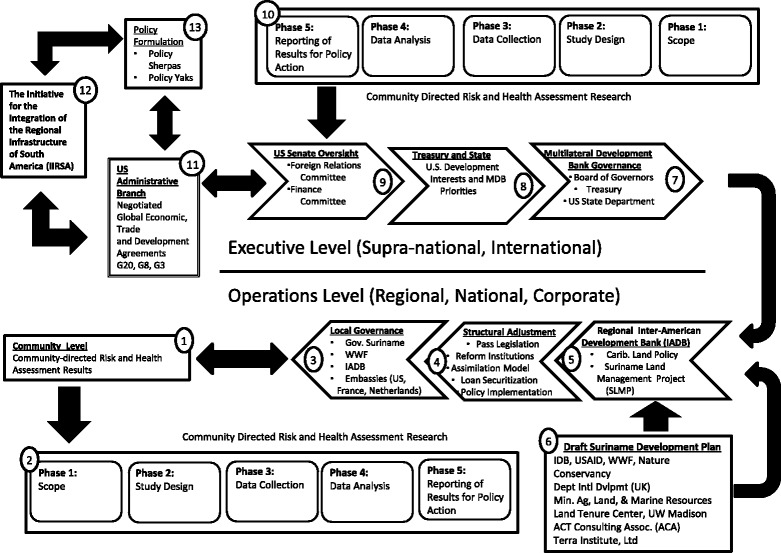
A *“Policy-Oriented Causal Diagram”* created to weave together the wide range of ideas and views captured during the interview process

Open-ended interviews were performed with participants listed in Table 2. The interviewer captured relevant stakeholders and relationships and added them to the Policy-Oriented Causal Diagram (Fig. 1). Open-ended interviews ensured that participant’s responses were not constrained by the interviewer and made it possible to explore the participant’s own knowledge structure.

One disadvantage of the method was that open-ended interviews were very time intensive. Also, the results were highly qualitative and not amenable to statistical analysis. Furthermore, the reliance on the interviewer to identify and extract important concepts and relationships increased the potential for bias and misjudgements. The diagram derived from the interviews, shown in in Fig. 1, was numerically keyed into 13 categories and described below:

### Community level (Fig. 1, Category 1)

Beginning in 2004, in response to early requests to collect hair samples, measure Hg and determine risk from exposure to Hg, indigenous community members in the Brokopondo and Sipaliwini districts in Suriname’s interior region pointed to the frequency with which the assessment of risk from exposure to Hg from mining had been conducted [19]. Indigenous individuals and communities were frustrated because the results were not made public and they were not benefitting from the work that was done. Indigenous communities recommended that researchers recognize the effects of ‘research pollution’, that is reticence, despair, mistrust and non-disclosure. Community leaders concluded that they wanted to determine for themselves whether they were at risk from exposure to Hg contamination and assess the potential health impacts from Hg exposure, especially in children. They requested assistance from the Suriname Indigenous Health Fund (SIHF), a non-profit non-governmental organization based in the United States, to provide technical expertise throughout the research process. SIHF provided a toxicologist, a sociologist, a physician and access to state-of-the-art portable Hg analyzer [28, 29]. Since 2004, five communities in Suriname requested to be included in the self-diagnosis research process. Those communities are listed in Table 2 (Nos. 1–5). In 2005, community sentiments favored economic development and, given the choice between “assimilate or else”, chose assimilation. By 2015, sentiments among communities undergoing assimilation noted that assimilation was not successful, government was not working for them, and they began asking, “what else is there”.

### Community directed risk and health assessment research (Fig. 1, Category 2)

Leaders of indigenous communities (Table 2, Nos. 1–5) determined they wanted to follow the example set by related Wayana communities in French Guiana and publish findings in an “internationally peer-reviewed journal” that would be acknowledged as legitimate by domestic and foreign government health care officials. After completing the self-directed research process between 2005 and 2012, participating communities and people living in voluntary isolation in the inter-riverine areas had possession of published data documenting the adverse health effects of Hg exposure and its link to gold mining [28–30]. One element of the community-led research process was that community-led research results were interpreted by the communities from their own cultural perspective prior to publication. They suggested that the policies and practices that permit the continued use of Hg in gold mining racially discriminate against tribal and indigenous people who subsist on contaminated fish.

### Local governance (Fig. 1, Category 3)

Beginning in 2007, attempts by the Government of Suriname (GOS) to suppress the results from the self-directed public health research on the impacts of Hg contamination from mining and economic development projects obstructed the intervention mapping process. Foreign health practitioners and in-country partners were warned of ‘dire consequences’ if they communicate the effects on public and environmental health from Hg contamination and more broadly from the structural adjustment programs (SAPs) being implemented in Suriname. The defenders of scientific censorship in Suriname claimed that research was the domain of experts who were an integral part of the SAP implementation process. Under this system, research became highly institutionalized through disciplines and fields of knowledge that were integral parts of the political and economic structures, funding agencies, universities, foreign NGOs, and development agencies that were implementing SAPs. It was the expressed position of these sector representatives that they had the right to set policy and deliver their an exclusive message in their own words.

### Structural adjustment (Fig. 1, Category 4)

During the implementation of the IADBs Suriname Land Management Project (SLMP), which coincided with the presidency of Ronald Venetiaan (2000–2010), the IADB addressed numerous issues that were constraining the efficient and effective allocation, use, and management of land and natural resources. The IADB determined that these issues needed to be addressed in a timely manner in order to transform resources into tradable commodities, develop Suriname’s economy, and reduce poverty. The SAP component of the SLMP was designed to align Suriname’s economy with global trade partners. The political system, legal framework, enforcement agencies, established patterns of social organization, public administration, and demographics of Suriname all form the country’s institutional base. These combine to make up Suriname’s economic structure that needed to be adapted in order to conform to the changing demands of investors. Various State and non-state actors explained that balance of payment requirements were what motivated Suriname to adopt adjustment policies that dispossessed indigenous people of their land and blocked access to their traditional sources of food and supplies they need for their livelihoods.

### Regional Inter-American Development Bank, IADB (Fig. 1, Category 5)

On October 15, 2013, a request was directed to the Suriname Country Office Representative of the IADB and copied to the Independent Consultation and Investigation Mechanism (ICIM) in which the negative impacts on health of indigenous communities due to gold-mining operations in the Interior region was described in detail [42, 43]. The purpose of the ICIM request was to initiate the Consultation Phase of the ICIM review process and address “the very difficult situation of the indigenous people who live in the villages of Puleowine (Apetina) as well as the inter-riverine ‘no-contact people’ living traditional lives in the forested regions of southeast Suriname”, as they are “being forced to abandon their minority cultural traits and merge with mainstream society. The petition went on to claim that the racial hostility they suffer and the lack of opportunity to participate in the central government or benefit adequately from resource distribution prevents the Wayana communities from becoming permanent and legitimate components of the society.” It was the stated purpose of the *Request* to address the structural impediments to public consultations of indigenous peoples.

The ICIM petition included an observation that representatives of the IADB presented the SLMP to the GOS on 3 March 2006. The SLMP contained a policy component on land tenure for the five Maroon and five Amerindian groups identified by the IADB. In addition to policy, the SLMP also outlined the legislation and regulations necessary for the GOS to implement the SLMP. It was noted, however, that legislation needed to be drafted and placed into law first in order for the policy to be implemented. The ICIM committee claimed that the Suriname Land Management Project [43], cited in the *Request*, was never approved or implemented and was taken off the pipeline in 2007. The Committee found, therefore, that the petition should be excluded from the Consultation process. Contradicting this finding was a written statement from IADB Operations Specialist at the Bank in Suriname, however, revealing that the SLMP had been completed and that 100% of the land and resources in Suriname’s Interior region had been concessioned. Also, a stated objective of the 2007 Project for the sustainable Development of the Interior was to include and implement the Suriname Land Management program [44].

The Request to the ICIM asked for assistance in determining whether action can be taken in either of two ways to address the situation facing indigenous people in Suriname who have been dispossessed of their land, impoverished and devastated by exposure to neurotoxic contaminants like Hg from mining: (1) by providing immediate relief at the community level to stabilize the situation over the short-term and (2) reducing death, disease and disability over the long-term by addressing the situation (i.e. structural violence) at the policy level. The Request also recommended the introduction of revisions to current structural adjustment programs for Suriname that will address the economic and public health challenges encountered in Suriname’s Interior region. The *Request* sought to engage empowered stakeholders relevant to the Suriname case to determine what conditions are necessary to bridge the divide between economic development and public health.

While the Eligibility Committee determined that the Request complied with the ‘submission criteria’, it also concluded it was not eligible for Consultation because it met ‘criteria for exclusion’. Specifically, requests dealing with a Bank-Financed Operation that are filed twenty-four (24) months after the last disbursement are excluded. As per the operation’s Transactions History Report, the last disbursement was made by the IADB to the Government of Suriname (GOS) on September 28, 2011, before closing the operation on October 7, 2011 whereas the ICIM/MICI petition was filed on October 15, 2013, 24 months plus 1 week after the operation closed. The eligibility Committee did acknowledge, however, that the request to the ICIM reasonably asserted that indigenous people in Suriname were materially and adversely affected by actions and omissions of the IADB in violation of a ‘relevant operational policy’ in a Bank-financed Operation.

Following the ICIM decision, IADB representatives acknowledged that the Bank is reluctant to address problems retrospectively if they are traceable to actions and omissions of projects that have been completed and for which funds have been disbursed. Although member states are reluctant to revisit completed projects they would be more likely to consider input from the public health sector if they were submitted before the project was approved, which is the basis for extending application of WHOs HiAP framework to the international level.

### Draft Suriname development plan (Fig. 1, Category 6)

Authors of the 2005 SLMP [43] were contracted by the IADB to perform an Environmental Impact Assessment (EIA) and a Health Impact Assessment (HIA). Both the EIA and HIA were used to assess the negative impacts of development projects, programs, and policies on the environment and on the health of the most vulnerable groups in Suriname. IADB mechanisms to ensure community and public participation in the project planning and development process were expressly designed to address controversy by giving community stakeholder groups an opportunity to vent their concerns about a project, “… in a way they perceive as just and fair and that ultimately gains public acceptance [45]”.

The authors of the SLMP acknowledged that the project would influence the health of indigenous people. Surprisingly, in spite of the fact that a concern for health was included in the discussion, health outcomes were not specifically considered nor were they given a high priority in the development plan [43]. The only actions taken to safeguard health under the SLMP plan was to inform local health authorities of planned development projects and to transfer the hidden costs of mitigating the causes of health and well-being to the health sector through the delivery of health services by the Ministry of Health (BOG) in Suriname. The economic development activities guided by the SLMP, which gave rise to the public health crisis affecting the lives of indigenous people in Suriname, do not include policy intervention strategies. These costs, which are externalized to Suriname’s Ministry of Health, and the private sector, do not take into account the people who become dispossessed and disassociated when land and resources are privatized and transferred to a tradeable market sector.

### Multilateral development bank governance (Fig. 1, Category 7)

Seeking high-level policy processes that would internalize the external public health costs of economic development programs, the IADB Board of Governors and the IADB president acknowledged that the biggest challenge the Bank faces in its approach to development and poverty reduction is in its ability to find business plans that meet the criteria of the commercial banking system [46]. A difficult challenge the Bank has not been able to address successfully is to find projects that meet its strategic goals of development with identity when they have political implications.

In 2015, SIHF partnered with a US treaty tribe to submit a proposal to the IADB Social Entrepreneurship Program (SEP). The SEP provides financing to disadvantaged populations and marginalized communities with interests in Value Chain enterprises. The proposal was made to expand a native-owned US-based forest product consortium to South America as a mechanism to support indigenous land title and resource management efforts in Suriname. The consortium would provide a pathway for tribal and indigenous people who are not currently in the global market economy to integrate. The proposal was based on a model that is a for-profit business that dedicates forest product revenues to social and environmental issues as well as shareholder value. The proposal was rejected because it did not meet the IADB’s emphasis on maximizing shareholder value and, therefore, did not meet the IADB’s eligibility criteria as an entrepreneurial project worthy of investment. Bank officials also said it would be necessary for tribal and indigenous people to spend time at the Bank in Washington, D.C. and familiarize themselves with the IADB and learn from the Bank how to submit a project proposal that meets Bank criteria [46].

### US Department of Treasury and State (Fig. 1, Category 8)

The community-led health assessment research took place in a complex social and political setting and it brought up many issues about relating to government officials, the media and communities when there existed a potential that study results could reflect poorly on government policies. Beginning in 2005, a campaign of intimidation and harassment of the SIHF public health team obstructed SIHF activities, its leadership and in-country partners.

The US State Department figured prominently in discussions regarding SIHF activities related to global health governance since it collaborates with the Treasury Department, promoted U.S. policy objectives, and advocated for U.S. economic and commercial interests in Suriname. Although the State Department did not make economic development policy nor intervene on behalf of individual public health practitioners or against the use of Hg in mining, it did promote US policy objectives and was responsible for bi-lateral relationships.

In contrast, the Treasury Department, which represents the US Administrative branch in the multilateral development banks and is dedicated to free market principles, emerged as the dominant policymaker with staff either in or dedicated to U.S. executive directors’ offices in six multilateral development banks, including the World Bank, Inter-American Development Bank, Asian Development Bank, the African Development Bank, and the European Bank for Reconstruction and Development, in which the US is a major shareholder [8, 47].

Although it is a goal of the U.S. Department of the Treasury, Office of Development Results and Accountability (ODRA) to engage with civil society and congressional staffers in order to provide oversight of safeguard policies, direct participation in high-level policy processes is discouraged. During a meeting in 2015, an ODRA representative was asked whether it would be possible to enhance current structural adjustment programs to address the persistent disjuncture between global health and governance as it relates to economic development and address the causes of health and well-being that lie outside the health sector and are socially and economically formed. Specifically, the Treasury was asked to talk about including provisions in SAP programs that would ameliorate the damaging effects of land privatization on the health and well-being of indigenous people that are dispossessed, dislocated, and impoverished by the process. In separate meetings, Treasury representatives were inconsistent in their opinion whether it was inappropriate to discuss the association of health and well-being of indigenous communities displaced and impoverished by economic development activities in an office of the Treasury Department, specifically the effects of land privatization projects and the securitization of development loans on the health of dispossessed communities.

### US senate oversight (Fig. 1, Category 9)

Since 2010, interviewers questioned why WHO’s Health in All Policies (HiAP) framework for country action had not been fully implemented in order to improve health outcomes. Senators from Washington State, California, and New Mexico and appropriate civil society coordinators at the Treasury Department and the IADB were contacted to discuss the full implementation of HiAP with a special emphasis on development bank policies and practices. Specific actions that could be taken within the context of the HiAP framework included, 1) request for a meeting with the Suriname government on behalf of indigenous communities undergoing forced assimilation; 2) investigate the possible use of sanctions against institutions responsible for gross violation of human rights. Our question was whether there is legislation would prohibit US (private commercial banks and US Treasury) from investing in economic development programs if a recipient country violates the human rights of its most vulnerable population; 3) contact and communicate with the appropriate office of the WHO to discuss the possibility of upgrading events in Suriname from ‘Ungraded’ (An event that requires no international response) to Grade 2 or 3 (A single or multiple country event with moderate or substantial public health consequences that requires a moderate or substantial international response); and 4) contact and communicate with the Pan-American Health Organization (PAHO) and the World Health Organization (WHO) to discuss the full implementation of the Health in all Policies framework at the international level with a special emphasis on development bank policies and practices.

### Community directed risk and health assessment research (Fig. 1, Category 10)

Since 2004, SIHF helped indigenous communities conduct research and document the lack of correspondence between progress in economic development and progress in social wellbeing among indigenous communities undergoing assimilation as a result of economic development. Phase 5 is the final step when results are reported for policy action. The most receptive audience for this information were legislators in the US Congress. From there, needs assessment information was disseminated to other agencies, most notably the Treasury Department.

Given that the community-led health assessment research took place in a complex social and political setting and that it brought up many issues about relating to government officials, the media and communities when there existed a potential that study results could reflect poorly on government policies, it became evident that there was no direct correlation between public health practice and global health governance. Instead, public health practice addressing social determinants of health led to political controversy instead of political accord. In order to proceed, SIHF practitioners had to abandon the idea that controversy caused by their work implied failure on the part of public health practitioners and had to accept controversy as a fundamental part of a democratic health intervention process. Since it is a fundamental feature of constitutional democracies to allow majority rule and, by definition, provide effective mechanisms for protecting minority rights, it became clear that public health practice in Suriname needed to operationalize WHO’s proposed ‘*Health in All Policies (HiAP) Framework for Country Action’* and identify specific methods that address the community and social health needs that accompany the economic development and assimilation processes [1].

### US administrative branch (Fig. 1, Category 11 and 12)

Free-trade agreements (FTAs), including the North American Free Trade Treaty, the Free Trade Area for the Americas, the Plan Colombia, the Regional Integration of Infrastructure in South America (IIRSA) and the Plan Puebla Panamá are the building blocks of the US policy for the Americas and the Caribbean. The key components of the FTAS are US and European stakeholders including hundreds of leading companies and banks in the US and EU [48]. FTAs are examples of international-level treaties that serve as the foundation for development policies in the Americas and the Caribbean. While US companies invest in FTAS, these economic plans cannot be disentangled from the political and security concerns of foreign missions. Instead, they work in tandem. The political system, legal framework, enforcement agencies, established patterns of social organization, public administration, and demographics of Suriname are all parts of economic structures that are adapted to conform to changing demands of global investors.

### Policy formation, multilateral financial cooperative (Fig. 1, Category 11 and 13)

Finance Ministers and Central Bank Governors from the Group of Twenty (G20) countries hold annual meetings to encourage policy coordination between advanced and emerging economies. Other governing bodies include the G7 (US, Japan, Germany, France, Great Britain, Italy, and Canada), and the BRICS (China, Brazil, India, Australia, and South Africa). Leaders of these governing bodies convene expert groups and decide when representatives of heads of state and ministers of finance, labor, agriculture, mining, and energy meet. Representatives of heads of state are tasked with coordinating the preparatory work involving the private sector. Although there is no central leadership, the leaders of six countries (US, Japan, China, Germany, France, and the UK) dominate and set global economic development objectives [46].

### Policy oriented causal diagram

The Policy-Oriented Causal Diagram that was developed (Fig. 1) is divided into two levels, the operations level (regional, national and corporate) and the executive level (supra-national, international) to delineate the boundry between domestic administrative law ‘of’ the States and international treaty law ‘between’ States. This legal dualism reflects a fundamental problem when the actions of international financial institutions regulated by treaties at the international level affect the health and well-being of people whose legal and democratic recourse is limited to the institutions of administrative law at the national level.

As discussions with stakeholders, which began at the community level and proceeded to higher levels on the Causal Diagram (Fig. 1), participants revealed a persistent disjuncture between the need to address the causes of negative impacts on health and well-being that are socially and economically formed and the inability of individuals within government to respond. Participants consistently reported their desire to help but their inability to respond due to constraints of their office and its corresponding mandate. As an alternative to direct action, IADB executives promoted entrepreneurialism as an adequate path to a global solution.

### Causal inference

Table 3 summarizes the indirect causal relationship between the health and well-being of indigenous Wayana people living in the southeast interior region of Suriname and specific economic development programs.

**Table 3:** Summary of the indirect causal relationship between the health and well-being of indigenous Wayana people living in the southeast interior region of Suriname and the Inter-American Development Bank's Suriname Land Management Project (SLMP) and the Sustainable Development of the Interior project (SU-T1026) using the five epidemiological criteria

Criteria	Metric	Outcome	Determination
Coherence	Cause and effect association supported by existing knowledge or theory	Existing knowledge and theory: Ref. Nos. [4–6, 16, 49, 51, 69–76]	Coherent
Strength of Association	Strong effect when exposed to cause	The main activity of multilateral investment funds is to provide loans for basic infrastructure projects and the conversion of non-marketed resources into the global market. Land and resource privatization occurs where indigenous people are living and confines them to plots of land that are too small for them to live, and where food sources are contaminated. Dispossessed indigenous people who survive become disabled, dislocated, scattered, impoverished and alienated minorities.	Strong
Time Order	Cause precedes effect in time	In 1958, the Surinamese government, within the context of the post-World War II Bretton-Woods global economic development plan, conducted Operation Grasshopper. The purpose of the project was a geological inventory of the interior region and the creation of economic opportunities for Suriname. Missionaries were the vanguards of economic development, land and resource privatization, and the displacement of indigenous people living in the area.	Cause Precedes Effect
Specificity	Effect traceable to single root cause	The current situation of Indigenous Peoples around the world is the result of a linear programme of “legal” precedent, originating with the Doctrine of Discovery and codified in contemporary national laws and policies [77]. Around the world, Indigenous Peoples are over-represented in all categories of disadvantage. In most indigenous communities people live in poverty without clean water and necessary infrastructure, lacking adequate health care, education, employment and housing. Many indigenous communities still suffer the effects of dispossession, forced removals from homelands and families, inter-generational trauma and racism, the effects of which are manifested in social welfare issues such as alcohol and drug problems, violence and social breakdown. Basic health outcomes dramatize the disparity in well-being between Indigenous Peoples and European descendants.	Effect Traceable to Single Root Cause
Consistency on Replication	Cause and effect association has been repeatedly observed by different persons, in different places, circumstances, and times	Ref. nos. of similar cause and effect associations observed: [4, 16, 26, 27]	Consistent

## Discussion

Getty writes that prior to colonization, indigenous peoples living traditional lifestyles were healthy, with organized systems of government, knowledge and kinship [48]. Although human welfare is conceptually complex and difficult to measure, academic literature tracing the health of societies undergoing assimilation is achieved by disciplines such as anthropology, archeology, economics, and history that offer broad perspectives and consider large-scale and long-term forces that shape community health. Steckel, for example, studied health and nutrition of people in pre-Columbian America who transitioned from hunter-gatherers to settled agriculture and from settled agriculture to urban lifestyles [49]. Using indicators of health from skeletal remains of people and a procedure for condensing diverse skeletal data into a single index that measured health and well-being, this cross-disciplinary study showed that at each stage of human development, a divergence occurred in which some people gained while others lost in the transitions. Data also reflected a trend in which the powerful had better nutrition, expended less energy on work, and were able to compel work among the less powerful. By the nineteenth century, urban living had a well-established reputation for poor health even as these areas continued to grow by in-migration. By 1839, Chadwick [50] and Morrissey [51] reported on the link between social stratification and mortality.

To provide a deeper analysis of fieldwork results, this paper also considered works in global health, law and sociology to inform the problem-solving process from a broad-based systems perspective as opposed to using simpler models that focus only on linear cause-and-effect pathways. The systems approach yields a social-ecological model in which health conditions can be viewed as a function of the interaction of individuals with the environment in which they live. A properly designed policy-oriented causal diagram would identify opportunities for making small changes in the environment that had the potential to lead to large improvements in health outcomes.

Environment as it pertains to the social determinants of health [52] can include family, social networks, political structures, and historical frameworks. In 1938, Burdick published a seminal work asserting that although the Roman Empire as a political organization passed away centuries ago, Roman jurisprudence, through its influence, still remains a world power [53]. In its modernized form, Roman Civil Law has been adapted and serves as the basis for the establishment of settlements, towns and cities and now is the basis of law for three-fourths of the economically developed world. Economic globalization, as we know it today, would not have been possible without the precedence of Roman Civil Law because it was designed to assimilate the indigenous occupants of seized territories after it was recognized that conquest and domination would not be sustainable.

In most instances, tribal peoples’ contact with the state and their assimilation into a broader economic community has been to their disadvantage [4, 5]. Just as new economic, political, social, and cultural relationships are being redefined by globalization in the 20^th^ and 21^st^ centuries, new patterns of morbidity and mortality are emerging. According to Durkheim [54], modern economic life produces a social pathology called anomie, which is the breakdown of social bonds between an individual and the community. Anomie is the thread that underlies psychosocial conditions like substance abuse and suicide. Durkheim speaks of the “rising flood of voluntary deaths” as “accompanying the march of civilization”. Durkheim also considered suicide rates to be a measure of the health the social body.

In Ecuador, Waters notes that conservative models of economic development demand decentralization and a weakening of the State to address the overlap of indigenous health and globalization and argues that participatory, community-based alternatives are more democratic [4]. Kunitz wrote that on the positive side, globalization and global information networks are making it possible for indigenous peoples to participate in global networks with other indigenous peoples, environmental and public health advocates, and non-governmental organizations to mobilize international support against adverse economic policies [6].

Although Suriname, a former Dutch colony, became politically independent in 1975, and its post-independence government was a socialist “friend of the underprivileged”, its development strategy was capitalist and similar to the strategy pursued during the colonial period [7]. Since Surinamese independence, there have been a succession of development plans that included the Multiannual Development Programme for 1975–1990 [7], the 1986 Agricultural and Trade Policy and Reform Act [15], UNDP’s Country Cooperation Framework of Suriname for 1997–2002 [14], the First Country Cooperation Framework for 1999–2001 [13], the Second Country Cooperation Framework for 2002–2006 [12], the 2002 Suriname Land Management Project [43], and the 2007 Project for the Sustainable Development of the Interior [44].

Evidence presented here suggests that neocolonial trade strategies in Suriname are having persistent and negative effects on communities of indigenous people when balance of payments requirements motivate the Suriname government to adopt adjustment policies that dispossess indigenous people of their land and livelihoods. It is an example of how the health and well-being of indigenous people is linked to inequalities generated by a neoliberal approach to economic development. This makes the issue of the relationship between economics, governance and health much broader than the Pan American Health Organization and World Health Organization 2012–2016 Country Cooperation Strategy for Suriname would suggest [55].

The PAHO/WHO strategy assesses health and health governance in the context of economic development and attempts to reconcile health with competing political and economic goals [55].

In Suriname, when health confronts World Bank, IADB and International Monetary Fund programs based on free trade and neoliberal economic globalization principles, the need for better health is evaluated in terms of the maximum amount of benefit for a given expenditure for various policy options and not in terms of the assumption that there is a basic right to health as expressed in the Alma Ata Declaration [56]. The Alma Ata Declaration states that, “health, which is a state of complete physical, mental and social wellbeing, and not merely the absence of disease or infirmity, is a fundamental human right [56]. In the field, however, health and well-being is a function of power rather than need, the innate characteristics of a particular health issue, and the compelling logic of an appropriate social action plan [56]”.

Economists who defend SAPs and IADB development projects say that providing special assistance would be a disincentive for indigenous communities who need to bootstrap their way into the economy. In contrast, indigenous communities undergoing assimilation are requesting land tenure so they can join the market economy and transform their land and resource assets into sustainable livelihoods. Land tenure is consistent with the market approach to economic development. It provides a means to compensate for the loss of resources according to the principles of eminent domain. If followed it would call for a restitution assistance program that would provide indigenous communities with resources they could use to bootstrap themselves into the Western economy.

## Conclusion

Globalization is leading the world to a culture united by consumerism and trade. At the same time the neoliberal model of globalization is resulting in a transfer of power to international banking institutions, a weakening of the nation state and the breakdown of local cultures. This has made local health institutions increasingly less able to respond to the public health demands inherent in the global economic development process. Suriname serves as a case example in which economic development programs result in an increase in GDP per capita at the expense of vulnerable minority tribal and indigenous people. These are people who reside in the areas where land and natural resources are being privatized and traded on the global market. Interventions within the arena of indigenous health necessarily have political implications. Attempts by the global health sector to reduce inequalities and social gradients to improve health and well-being of indigenous people have to contend with the political, economic and cultural realities of consumer capitalism and globalization.

Economic development projects like the SLMP in Suriname lacked distinct mechanisms that are ethnically, socially, or culturally appropriate despite the fact that they implicitly include indigenous peoples among the beneficiaries. In practice, they failed to benefit and ultimately harmed the indigenous segment of the target population. A simple trade-off between benefits for a majority and the devastating effects for a minority is unacceptable. The sustainability of development can be ensured only if unacceptable harm to vulnerable minorities is avoided, the full range of potential impacts are assessed at an early stage, and action is taken in light of the outcome of the assessment.

The argument that the global economic community has a special moral obligation to indigenous people harmed by multilateral investments in global economic development projects has practical implications for many areas of social policy, including high-level health care policy. It justifies isolating expenditures on indigenous health initiatives from general funding limits. It also justifies special expenditures on indigenous health programs designed to address the unique needs of indigenous people in remote, sparsely populated areas. Providing special resources would contribute to overcoming large health inequalities.

Health challenges currently experienced by indigenous communities in Suriname can only be tackled by radical changes to the way in which global health and financial and economic governance are currently managed. It is clear that decades of public health intervention among indigenous communities in Suriname has neglected to include a social action plan as it relates to these already marginalized communities. We suggest here that the goal of the global public health sector should be to advance UNDPs expressed “people-centered” strategies for economic-development that simultaneously meet the demands of conservative neoliberal economic development models as well as democratic participatory, community-based development models that also meet the minimum standards for health and human rights. We expect the lessons learned in Suriname can be replicated in other areas experiencing similar issues.

We conclude with three recommendations for improving Suriname’s compliance with its domestic and international commitments and obligations related to the effects of economic development projects and Indigenous Peoples’ right to health. These recommendations are replicable and should facilitate priority setting in other countries with great disparities in health experienced by Indigenous Peoples or other disadvantaged population groups.

### Recommendation #1

Establish improved social services, and translate gains in the macro-economic sphere into concrete benefits for the entire population with a special emphasis on the most vulnerable groups, we recommend that Suriname engage and work with its foreign partners, including foreign Development Banks, with international NGOs, powerful concession holders and aligned foreign governments to recast the Structural Adjustment Programs to provide land tenure to indigenous communities so communities undergoing assimilation and acculturation can join the market economy and transform their land assets into sustainable livelihoods. This recommendation is in accordance with existing international financial institution policies and strategies and the UNDPs people-centered development strategy that includes improved governance and poverty alleviation and sustainable livelihood generation with a specific focus on the interior,

In Suriname, the inability to reconcile health with competing priorities and political and economic goals is evidence that while health has been liberalized, commodified and marketized, the marketplace is not an inevitable agent of progress because it preferentially responds to competing economic interests, the demands of wealth and the operation of power rather than need, or the innate characteristics of a particular health issue or the compelling logic of a public health interventnion plan.

### Recommendation #2

Advocates for indigenous health in Suriname should demand that the World Health Organization upgrade the public health emergency status in Suriname from ‘Ungraded’ (An event that requires no international response) to Grade 3 (A multiple country event including French Guiana and Brazil with substantial public health consequences that requires a substantial international response);

### Recommendation #3

The World Health Organization should fully implement its *Health in all Policies (HiAP) framework for Country Action* at the international level with a special emphasis on high-level development policies applied to Suriname that guide development bank practices (e.g., Inter-American Development Bank, World Bank). The Government of Suriname should demand that international development policies and programs internalize the external social and health costs of development affecting Suriname’s most vulnerable groups.

## Abbreviations

CCF: Country Cooperation Framework
EIA: Environmental Impact Assessment
FDI: Foreign Direct Investment
FTA: Free-Trade Agreement
G20: Group of 20 Nations
GDP: Gross Domestic Product
GOS: Government of Suriname
Hg: Mercury
HIA: Health Impact Assessment
HiAP: WHO’s Health in All Policies (HiAP) Framework for Country Action
IADB: Inter-American Development Bank
ICIM: Independent Consultation and Investigation Mechanism
IIRSA: Regional Integration of Infrastructure in South America
MDB: Multi-Lateral Development Bank
ODRA: U.S. Department of the Treasury, Office of Development Results and Accountability
OIM: Outside Initiative Model
PAHO: Pan-American Health Organization
SAP: Structural Adjustment Program
SEP: Social Entrepreneurship Program
SIHF: Suriname Indigenous Health Fund
SLMP: Suriname Land Management Project
WHO: World Health Organization
